# 4-(6-Chloro­imidazo[1,2-*b*]pyridazin-3-yl)benzonitrile

**DOI:** 10.1107/S1600536811037901

**Published:** 2011-09-30

**Authors:** Yiliang Zhao, Clarissa K. L. Ng

**Affiliations:** a Faculty of Pharmacy, Science Road, The University of Sydney, Sydney, Australia

## Abstract

In the title compound, C_13_H_7_ClN_4_, the imidazopyridazine ring system is essentially planar [maximum deviation 0.015 (1) Å]. It is inclined to the benzene ring of the benzonitrile group by 11.31 (2)°. In the crystal, molecules are linked *via* C—H⋯Cl and C—H⋯N interactions.

## Related literature

For related structures, see Kia *et al.* (2009 ▶); Khan *et al.* (2010 ▶); Xue (2010 ▶); Zhao *et al.* (2009 ▶).
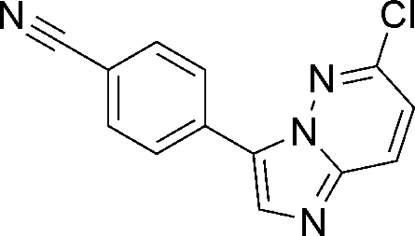


## Experimental

### 

#### Crystal data

C_13_H_7_ClN_4_
*M* *_r_* = 254.68
Tetragonal, 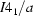

*a* = 13.5513 (12) Å
*c* = 24.566 (3) Å
*V* = 4511.3 (7) Å^3^
*Z* = 16
Mo *K*α radiation
μ = 0.32 mm^−1^
*T* = 150 K
0.25 × 0.15 × 0.10 mm

#### Data collection

Bruker SMART APEXII CCD diffractometer
Absorption correction: multi-scan (*SADABS*; Bruker, 2008 ▶) *T*_min_ = 0.659, *T*_max_ = 0.746
140841 measured reflections
18107 independent reflections
11833 reflections with *I* > 2σ(*I*)
*R*_int_ = 0.032

#### Refinement

*R*[*F* ^2^ > 2σ(*F* ^2^)] = 0.043
*wR*(*F* ^2^) = 0.141
*S* = 1.08
18107 reflections
191 parameters
7 restraints
All H-atom parameters refined
Δρ_max_ = 0.85 e Å^−3^
Δρ_min_ = −0.34 e Å^−3^

### 

Data collection: *APEX2* (Bruker, 2008 ▶); cell refinement: *SAINT* (Bruker, 2008 ▶); data reduction: *SAINT*; program(s) used to solve structure: *SHELXS97* (Sheldrick, 2008 ▶); program(s) used to refine structure: *SHELXL97* (Sheldrick, 2008 ▶); molecular graphics: *ORTEP-3 for Windows* (Farrugia, 1999 ▶); software used to prepare material for publication: *SHELXL97* and *publCIF* (Westrip, 2010 ▶).

## Supplementary Material

Crystal structure: contains datablock(s) I, global. DOI: 10.1107/S1600536811037901/ng5227sup1.cif

Structure factors: contains datablock(s) I. DOI: 10.1107/S1600536811037901/ng5227Isup2.hkl

Supplementary material file. DOI: 10.1107/S1600536811037901/ng5227Isup3.cml

Additional supplementary materials:  crystallographic information; 3D view; checkCIF report

## Figures and Tables

**Table 1:** Hydrogen-bond geometry (Å, °)

*D*—H⋯*A*	*D*—H	H⋯*A*	*D*⋯*A*	*D*—H⋯*A*
C2—H2⋯N1^i^	1.08 (1)	2.60 (1)	3.3502 (7)	126 (1)
C3—H3⋯Cl1^ii^	1.08 (1)	2.70 (1)	3.7389 (6)	161 (1)
C5—H5⋯N4^iii^	1.08 (1)	2.31 (1)	3.3341 (7)	157 (1)
C8—H8⋯N4^iii^	1.08 (1)	2.52 (1)	3.6049 (8)	177 (1)
C12—H12⋯N2	1.08 (1)	2.25 (1)	2.9975 (6)	125 (1)

Symmetry codes: (i) 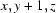; (ii) 
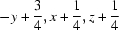
; (iii) 
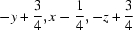
.

**Fig. 1.**
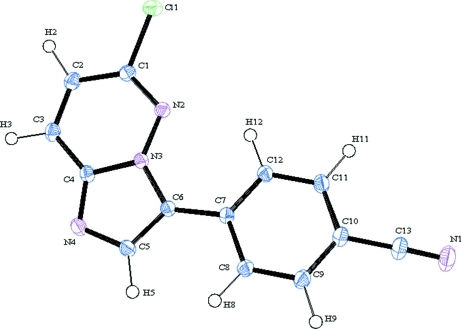
, The ORTEP view of the title compound with 50% probability displacement elipsoids for non-H atoms.

## supplementary crystallographic information

### Comment

The title compound, C_13_ H_7_ C_l1_ N_4_, was newly synthesized, crystallized and analysed at high resolution sin theta/lambda 1.1. The bond lengths are as expected. The crystal packing reveals that in one unit cell, every four molecules stack in parallel position by a number of non-classical hydrogen bonds. Hydrogen atoms are fixed at 1.083 A as determined from neutron diffraction, and the temperature factors were refined isotropically.

### Experimental

To a thick wall borosilicate glass vial were added 6-Chloro-3-iodo-imidazo[1,2-*b*]pyridazine (0.05 g, 1.0 equiv.), 4-cyanophenylboronic acid (0.032 g, 1.2 equiv.), Pd(PPh_3_)_4_ (0.02 g, 10 mol%), K_2_CO_3_ (0.037 g, 1.5 equiv.), and EtOH/H2O (4 : 1, 5 ml). The vial was sealed with a silicon septum and the reaction mixture was pre-stirred for 1 min. The reaction mixture was subjected to microwave irradiation at 90 °C for 14 min at the maximum power of 300 W with sufficient stirring. The reaction mixture was cooled to room temperature and solvent was removed under reduced pressure. The crude product was purified by flash column chromatography, eluting with ethyl acetate/hexane (1 : 1) to give the titled compound as a bright yellow solid (0.038 g, 83 %).

### Crystal data

**Table:**

C_13_H_7_ClN_4_	*D*_x_ = 1.500 Mg m^−^^3^
*M**_r_* = 254.68	Mo *K*α radiation, λ = 0.71073 Å
Tetragonal, *I*4_1_/*a*	Cell parameters from 500 reflections
*a* = 13.5513 (12) Å	θ = 1.7–62.3°
*c* = 24.566 (3) Å	µ = 0.32 mm^−^^1^
*V* = 4511.3 (7) Å^3^	*T* = 150 K
*Z* = 16	Block, colourless
*F*(000) = 2080	0.25 × 0.15 × 0.10 mm
none	

### Data collection

**Table:**

Bruker SMART APEXII CCD diffractometer	18107 independent reflections
Radiation source: fine-focus sealed tube	11833 reflections with *I* > 2σ(*I*)
graphite	*R*_int_ = 0.032
ω scans	θ_max_ = 62.3°, θ_min_ = 1.7°
Absorption correction: multi-scan (*SAINT*; Bruker, 2008)	*h* = −33→32
*T*_min_ = 0.659, *T*_max_ = 0.746	*k* = −33→31
140841 measured reflections	*l* = −58→60

### Refinement

**Table:**

Refinement on *F*^2^	Primary atom site location: structure-invariant direct methods
Least-squares matrix: full	Secondary atom site location: difference Fourier map
*R*[*F*^2^ > 2σ(*F*^2^)] = 0.043	Hydrogen site location: inferred from neighbouring sites
*wR*(*F*^2^) = 0.141	All H-atom parameters refined
*S* = 1.08	*w* = 1/[σ^2^(*F*_o_^2^) + (0.0756*P*)^2^] where *P* = (*F*_o_^2^ + 2*F*_c_^2^)/3
18107 reflections	(Δ/σ)_max_ = 0.001
191 parameters	Δρ_max_ = 0.85 e Å^−^^3^
7 restraints	Δρ_min_ = −0.34 e Å^−^^3^

### Special details

**Table:**

Geometry. All e.s.d.'s (except the e.s.d. in the dihedral angle between two l.s. planes) are estimated using the full covariance matrix. The cell e.s.d.'s are taken into account individually in the estimation of e.s.d.'s in distances, angles and torsion angles; correlations between e.s.d.'s in cell parameters are only used when they are defined by crystal symmetry. An approximate (isotropic) treatment of cell e.s.d.'s is used for estimating e.s.d.'s involving l.s. planes.
Refinement. Refinement of *F*^2^ against ALL reflections. The weighted *R*-factor *wR* and goodness of fit *S* are based on *F*^2^, conventional *R*-factors *R* are based on *F*, with *F* set to zero for negative *F*^2^. The threshold expression of *F*^2^ > σ(*F*^2^) is used only for calculating *R*-factors(gt) *etc*. and is not relevant to the choice of reflections for refinement. *R*-factors based on *F*^2^ are statistically about twice as large as those based on *F*, and *R*- factors based on ALL data will be even larger.Reflections -1 11 4, -2 4 0, -5 7 10, 4 4 0, 2 4 2, 1 8 13, -4 8 6, 1 3 16, -2 4 22, -1 10 1, -1 6 1, 0 0 24, -4 6 10, 4 5 11, -2 5 7 were omitted due to bad agreement statistics.

### Fractional atomic coordinates and isotropic or equivalent isotropic displacement parameters (Å^2^)

**Table:**

	*x*	*y*	*z*	*U*_iso_*/*U*_eq_	
C1	0.39217 (3)	0.36640 (3)	0.159032 (17)	0.01678 (5)	
C2	0.37908 (3)	0.45664 (3)	0.187490 (19)	0.01942 (6)	
C3	0.36436 (3)	0.45225 (3)	0.242477 (19)	0.01919 (6)	
C4	0.36372 (3)	0.35883 (3)	0.267513 (16)	0.01652 (5)	
C5	0.35942 (3)	0.23262 (3)	0.319699 (16)	0.01807 (5)	
C6	0.37353 (3)	0.19353 (3)	0.267716 (15)	0.01514 (5)	
C7	0.37986 (3)	0.09040 (3)	0.251063 (15)	0.01514 (5)	
C8	0.38792 (3)	0.01863 (3)	0.292379 (18)	0.01890 (6)	
C9	0.38734 (4)	−0.08101 (3)	0.27984 (2)	0.02124 (7)	
C10	0.38037 (3)	−0.11125 (3)	0.22543 (2)	0.01965 (6)	
C11	0.37593 (3)	−0.04085 (3)	0.183904 (18)	0.01980 (6)	
C12	0.37476 (3)	0.05927 (3)	0.196657 (16)	0.01772 (5)	
C13	0.37580 (4)	−0.21460 (3)	0.21251 (2)	0.02413 (8)	
N1	0.37014 (5)	−0.29780 (4)	0.20267 (3)	0.03142 (10)	
N2	0.39085 (2)	0.27773 (2)	0.179828 (13)	0.01557 (4)	
N3	0.37627 (2)	0.27594 (2)	0.234420 (13)	0.01445 (4)	
N4	0.35305 (3)	0.33273 (3)	0.319431 (15)	0.01907 (5)	
Cl1	0.413952 (9)	0.370824 (9)	0.089725 (5)	0.02143 (3)	
H2	0.3846 (8)	0.5243 (4)	0.1643 (3)	0.031 (2)*	
H3	0.3542 (7)	0.5204 (4)	0.2647 (4)	0.030 (2)*	
H5	0.3569 (8)	0.1898 (7)	0.3569 (2)	0.034 (3)*	
H8	0.3999 (8)	0.0435 (7)	0.33372 (15)	0.031 (2)*	
H9	0.3914 (7)	−0.1341 (6)	0.3127 (3)	0.029 (2)*	
H11	0.3662 (8)	−0.0628 (9)	0.14188 (16)	0.042 (3)*	
H12	0.3662 (7)	0.1155 (5)	0.1657 (3)	0.030 (2)*	

### Atomic displacement parameters (Å^2^)

**Table:**

	*U*^11^	*U*^22^	*U*^33^	*U*^12^	*U*^13^	*U*^23^
C1	0.01593 (12)	0.01674 (12)	0.01767 (13)	0.00052 (9)	−0.00065 (9)	0.00152 (9)
C2	0.02065 (14)	0.01551 (12)	0.02209 (15)	0.00112 (10)	−0.00169 (11)	0.00123 (10)
C3	0.02072 (14)	0.01484 (12)	0.02199 (15)	0.00197 (10)	−0.00191 (11)	−0.00164 (10)
C4	0.01706 (12)	0.01544 (11)	0.01706 (12)	0.00196 (9)	−0.00169 (9)	−0.00230 (9)
C5	0.02051 (14)	0.01867 (13)	0.01504 (11)	0.00221 (10)	−0.00118 (10)	−0.00095 (9)
C6	0.01548 (11)	0.01474 (11)	0.01520 (11)	0.00104 (8)	−0.00108 (8)	−0.00048 (8)
C7	0.01486 (11)	0.01468 (11)	0.01587 (11)	0.00048 (8)	−0.00133 (8)	−0.00043 (8)
C8	0.02312 (15)	0.01589 (12)	0.01768 (13)	0.00058 (10)	−0.00269 (11)	0.00081 (10)
C9	0.02647 (18)	0.01571 (13)	0.02155 (15)	0.00072 (11)	−0.00397 (13)	0.00104 (11)
C10	0.02066 (14)	0.01475 (12)	0.02353 (16)	0.00046 (10)	−0.00355 (12)	−0.00161 (11)
C11	0.02297 (16)	0.01711 (13)	0.01932 (14)	−0.00005 (11)	−0.00236 (11)	−0.00292 (10)
C12	0.02068 (14)	0.01622 (12)	0.01625 (12)	−0.00001 (10)	−0.00164 (10)	−0.00097 (9)
C13	0.02702 (19)	0.01604 (14)	0.0293 (2)	0.00087 (12)	−0.00495 (16)	−0.00242 (13)
N1	0.0399 (3)	0.01696 (15)	0.0374 (3)	0.00031 (15)	−0.0067 (2)	−0.00451 (15)
N2	0.01569 (10)	0.01588 (10)	0.01514 (9)	0.00048 (7)	−0.00012 (7)	0.00018 (7)
N3	0.01422 (9)	0.01436 (9)	0.01476 (9)	0.00097 (7)	−0.00081 (7)	−0.00084 (7)
N4	0.02264 (13)	0.01842 (12)	0.01614 (11)	0.00297 (9)	−0.00162 (9)	−0.00302 (9)
Cl1	0.02372 (5)	0.02314 (5)	0.01744 (4)	0.00047 (3)	0.00083 (3)	0.00373 (3)

### Geometric parameters (Å, °)

**Table:**

C1—N2	1.3058 (5)	C7—C12	1.4033 (5)
C1—C2	1.4197 (6)	C7—C8	1.4100 (6)
C1—Cl1	1.7291 (5)	C8—C9	1.3849 (6)
C2—C3	1.3668 (7)	C8—H8	1.0824 (10)
C2—H2	1.0819 (10)	C9—C10	1.4014 (7)
C3—C4	1.4074 (6)	C9—H9	1.0824 (10)
C3—H3	1.0816 (10)	C10—C11	1.3980 (7)
C4—N4	1.3315 (6)	C10—C13	1.4374 (6)
C4—N3	1.3970 (5)	C11—C12	1.3925 (6)
C5—N4	1.3593 (6)	C11—H11	1.0825 (10)
C5—C6	1.3957 (5)	C12—H12	1.0823 (10)
C5—H5	1.0827 (10)	C13—N1	1.1557 (7)
C6—N3	1.3847 (5)	N2—N3	1.3558 (5)
C6—C7	1.4588 (5)		
			
N2—C1—C2	126.74 (4)	C9—C8—C7	120.80 (4)
N2—C1—Cl1	114.80 (3)	C9—C8—H8	120.9 (6)
C2—C1—Cl1	118.45 (3)	C7—C8—H8	118.2 (6)
C3—C2—C1	117.87 (4)	C8—C9—C10	119.84 (4)
C3—C2—H2	124.6 (5)	C8—C9—H9	118.8 (6)
C1—C2—H2	117.5 (5)	C10—C9—H9	121.4 (6)
C2—C3—C4	118.16 (4)	C11—C10—C9	119.96 (4)
C2—C3—H3	118.7 (5)	C11—C10—C13	120.14 (4)
C4—C3—H3	123.2 (5)	C9—C10—C13	119.89 (4)
N4—C4—N3	110.92 (3)	C12—C11—C10	120.08 (4)
N4—C4—C3	131.17 (4)	C12—C11—H11	118.7 (7)
N3—C4—C3	117.91 (4)	C10—C11—H11	120.9 (7)
N4—C5—C6	112.52 (4)	C11—C12—C7	120.44 (4)
N4—C5—H5	122.4 (6)	C11—C12—H12	121.9 (5)
C6—C5—H5	125.0 (6)	C7—C12—H12	117.6 (5)
N3—C6—C5	103.77 (3)	N1—C13—C10	178.50 (7)
N3—C6—C7	127.26 (3)	C1—N2—N3	113.92 (3)
C5—C6—C7	128.92 (4)	N2—N3—C6	127.06 (3)
C12—C7—C8	118.82 (4)	N2—N3—C4	125.38 (3)
C12—C7—C6	123.52 (3)	C6—N3—C4	107.54 (3)
C8—C7—C6	117.62 (3)	C4—N4—C5	105.24 (3)
			
N2—C1—C2—C3	0.66 (7)	C8—C7—C12—C11	1.11 (6)
Cl1—C1—C2—C3	−178.41 (3)	C6—C7—C12—C11	−176.50 (4)
C1—C2—C3—C4	0.41 (6)	C11—C10—C13—N1	−112 (3)
C2—C3—C4—N4	178.65 (4)	C9—C10—C13—N1	67 (3)
C2—C3—C4—N3	−1.24 (6)	C2—C1—N2—N3	−0.74 (6)
N4—C5—C6—N3	0.34 (5)	Cl1—C1—N2—N3	178.35 (3)
N4—C5—C6—C7	−177.28 (4)	C1—N2—N3—C6	−178.44 (4)
N3—C6—C7—C12	−10.13 (6)	C1—N2—N3—C4	−0.23 (5)
C5—C6—C7—C12	166.96 (4)	C5—C6—N3—N2	178.38 (3)
N3—C6—C7—C8	172.24 (4)	C7—C6—N3—N2	−3.94 (6)
C5—C6—C7—C8	−10.67 (6)	C5—C6—N3—C4	−0.09 (4)
C12—C7—C8—C9	−2.26 (6)	C7—C6—N3—C4	177.58 (4)
C6—C7—C8—C9	175.48 (4)	N4—C4—N3—N2	−178.70 (4)
C7—C8—C9—C10	1.11 (7)	C3—C4—N3—N2	1.21 (6)
C8—C9—C10—C11	1.22 (7)	N4—C4—N3—C6	−0.19 (4)
C8—C9—C10—C13	−177.53 (5)	C3—C4—N3—C6	179.72 (4)
C9—C10—C11—C12	−2.37 (7)	N3—C4—N4—C5	0.39 (5)
C13—C10—C11—C12	176.38 (4)	C3—C4—N4—C5	−179.51 (4)
C10—C11—C12—C7	1.19 (7)	C6—C5—N4—C4	−0.46 (5)

### Hydrogen-bond geometry (Å, °)

**Table:**

*D*—H···*A*	*D*—H	H···*A*	*D*···*A*	*D*—H···*A*
C2—H2···N1^i^	1.08 (1)	2.60 (1)	3.3502 (7)	126.(1)
C3—H3···Cl1^ii^	1.08 (1)	2.70 (1)	3.7389 (6)	161.(1)
C5—H5···N4^iii^	1.08 (1)	2.31 (1)	3.3341 (7)	157.(1)
C8—H8···N4^iii^	1.08 (1)	2.52 (1)	3.6049 (8)	177.(1)
C12—H12···N2	1.08 (1)	2.25 (1)	2.9975 (6)	125.(1)

Symmetry codes: (i) *x*, *y*+1, *z*; (ii) −*y*+3/4, *x*+1/4, *z*+1/4; (iii) −*y*+3/4, *x*−1/4, −*z*+3/4.
